# A malignant omental extra-gastrointestinal stromal tumor on a young man: a case report and review of the literature

**DOI:** 10.1186/1477-7819-6-50

**Published:** 2008-05-15

**Authors:** Mario Castillo-Sang, Salim Mancho, Albert W Tsang, Barbu Gociman, Babatunde Almaroof, Mohammed Y Ahmed

**Affiliations:** 1 Department of Surgery, The University of Toledo Health Science Campus Toledo, Ohio, USA; 2 Division of Trauma Surgery, Department of Surgery. Saint Vincent Mercy Medical Center, Toledo, Ohio, USA

## Abstract

**Background:**

Gastrointestinal stromal tumors (GIST) are uncommon intra-abdominal tumors. These tumors tend to present with higher frequency in the stomach and small bowel. In fewer than 5% of cases, they originate primarily from the mesentery, omentum, or peritoneum. Furthermore, these extra-gastrointestinal tumors (EGIST) tend to be more common in patients greater than 50 years of age. Rarely do EGIST tumors present in those younger than 40 years of age.

**Case presentation:**

We report a case of a large EGIST in a 27-year-old male. An abdominal pelvic computerized tomography imaging demonstrated an intra-abdominal mass of 22 cm, without invasion of adjacent viscera or liver lesions. This mass was resected *en bloc *with its fused omentum and an adherent portion of sigmoid colon. Pathology results demonstrated a malignant gastrointestinal stromal tumor with positive CD117 (c-kit) staining, and negative margins of resection, and no continuity of tumor with the sigmoid colon. Due to the malignant and aggressive nature of this patient's tumor, he was started on STI-571 as adjuvant chemotherapy.

**Conclusion:**

Stromal tumors of an extra-gastrointestinal origin are rare. Of the reported omental and mesenteric EGISTs in four published series, a total of 99 tumors were studied. Of the 99 patients in these series only 8 were under 40 years of age, none were younger than 30 years old; and only 5 were younger than 35 years old. Our patient's age is at the lower end of the age spectrum for the reported EGISTs. Young patients who present with an extra-gastrointestinal stromal tumor (EGIST), who have complete resection with negative margins, have a good prognosis. There is little data to support the role of STI-571 in adjuvant or neoadjuvant therapy after curative resection. Given the lack of data, the use of STI-571 must be individualized.

## Background

Gastrointestinal stromal tumors (GIST) are the most common mesenchymal tumors of the gastrointestinal tract, although their overall incidence is low. In the United States of America, it is estimated that 3,300 to 4,350 new GISTS are diagnosed every year. It is well accepted that their cell of origin is the interstitial cell of Cajal. The commonality of these tumors is a positive immunohistochemistry stain to CD117 also known as C-kit located at 4q11-12. Mutations have frequently been identified in exon 11 of the C-kit gene, but also on exons 9 and 13 [1,2]. These tumors also tend to be positive for CD34 [3]. Tumors previously diagnosed as gastrointestinal leiomyomas, leiomyoblastomas, and leiomyosarcomas, as well as tumors previously deemed neurofibromas or schwannomas [2] are now re-classified as GISTs based on immunohistochemistry.

Gastrointestinal stromal tumors can appear anywhere in the gastrointestinal tract, from the mouth to the anus, but also in extra-gastrointestinal locations such as mesentery, omentum, peritoneum [4-6]. Gastrointestinal stromal tumors arise more commonly found in the stomach (40–70%), small intestine (20–40%), and colon (5–15%). Omental, mesenteric, and retroperitoneal tumors comprise less than 5% [1,2,5,7]. The independent predicting factors of tumor behavior are tumor size and mitotic activity [2,5,7-9], but age and location are also predictive factors [10]. The significance of tumor site in prediction of malignant behavior is site dependant [7]. Gastrointestinal stromal tumors are a disease entity predominantly of people older than 50 years of age, with adults less than 40 years of age accounting for 5% to 20% [2]. Children account for less than 3% of GIST [1,11].

## Case presentation

We present the case of a 27 years old Caucasian male that presented to our emergency department with chief complaint of right lower quadrant abdominal pain. The patient was referred with a 24 hour onset of colicky pain, of seven of ten in intensity. He denied bowel habit changes, fevers or chills, but he did complain of nausea, and postprandial fullness, but no vomiting. There was no history of weight loss over the last year and he denied any abdominal trauma or past surgeries. His family history was significant for colonic cancer in his father at age 47 and breast cancer in his mother at age 60.

Examination showed a well-developed male in no acute distress with obvious abdominal distension. Abdominal palpation demonstrated a large mass extending from the right upper quadrant and epigastrium to the right lower quadrant. The mass was non-pulsatile, moderately tender, and non-mobile. No peritoneal signs were appreciated. His lower extremities showed no edema, and his rectal examination was negative for masses or gross or microscopic blood in the stools.

**Figure 1:**
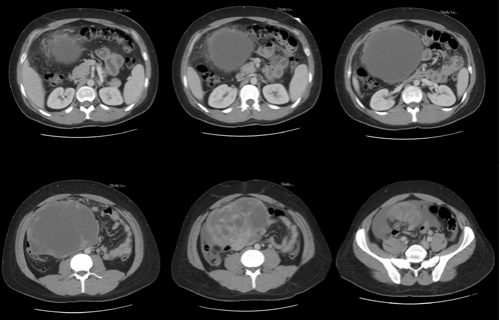
CT images at six different levels demonstrates a large 22 cm intraabdominal mass displacing the small bowel to the left.

A computerized tomography of the abdomen and pelvis was performed which showed a large ovoid intraabdominal heterogeneous mass measuring 22 cm in greatest length extending from the right upper quadrant to the pelvis without invasion into adjacent viscera (Fig 1). Chest X-ray showed no lung parenchyma or bony lesions, and the CT scan of the abdomen was negative for liver lesions. A colonoscopy was attempted, but we were unable to pass the transverse colon due to the extraluminal compression, but no polyps, lesions or blood were appreciated. Tumor markers were drawn for CA-19-9, CA-125, beta-HCG, and alpha-fetoprotein. Only CA-123 was elevated at 128.

**Figure 2:**
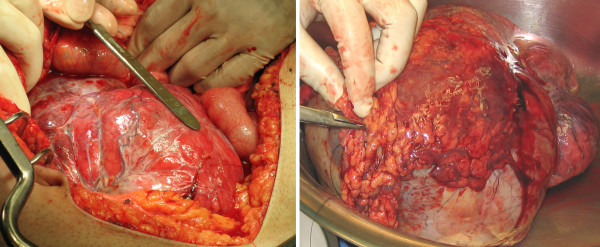
**Intraoperative images show a large mass within the abdomen and the displacement of the small bowel (left).** The mass originated from the greater omentum as can be seen on the right.

The patient was operated for resection of the mass. Upon gaining access to the peritoneal cavity, a moderate amount of serosanguinous fluid was evident and collected for cytology. A "muscular" mass was immediately apparent and occupied most of the peritoneal cavity (Fig 2). The mass arose from the greater omentum, which was densely fused to it. On its inferior pole the mass was in close apposition to the sigmoid colon, but was not fused to it.

The mass was removed *en block *with the greater omentum and the adjacent sigmoid colon. The margins were sent for frozen section and came back as negative for malignancy. Cytology of the peritoneal fluid revealed reactive mesothelial cells.

**Figure 3:**
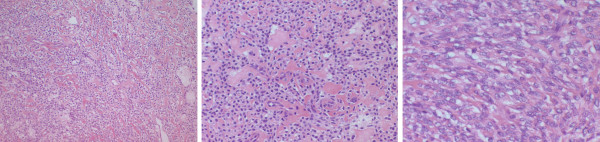
Microscopic imaging of the tumor at 10×, 20×, 40× shown with H&E staining.

**Figure 4:**
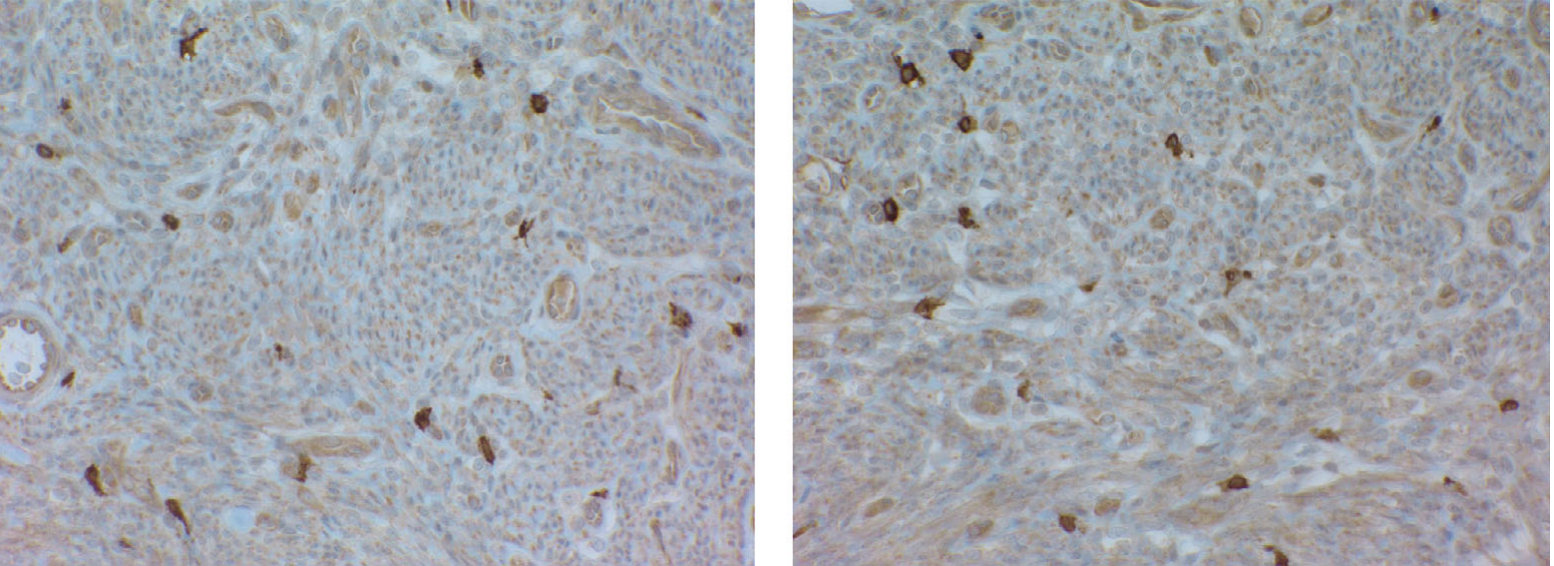
The tumor showed strong positivity to CD117 staining.

Final pathology showed a malignant gastrointestinal stromal tumor with smooth muscle differentiation and negative margins of resection. Based on the size of the neoplasm and necrosis present, in spite of few mitoses, this tumor was best viewed as a malignant or at least an aggressive extra-gastrointestinal stromal tumor (Fig 3). Immunostainings for LCA, CD117, CD34, pancytokeratin, s100, smooth muscle actin, calretinin, EMA, vimentin, CEA, LEU M1, and Factor VIII were done. The positive results included CD 117 (C-Kit), smooth muscle actin and vimentin (Fig 4). The postoperative period was uncomplicated, and medical oncology service was consulted and the patient was placed on a STI-571 regimen.

## Discussion

The extra-gastrointestinal stromal tumors (EGIST) studied by Miettinen *et al*., [4] (13 omental and 10 mesenteric) showed low mitotic activity. They were typically positive for CD117, but less so for CD34. Like our case, these EGIST often showed alpha smooth muscle actin reactivity, but were all negative for desmin and S-100 protein [4].

The reported cases of extra-gastrointestinal stromal tumors (EGIST) have included omental, mesenteric, and retroperitoneal tumors. The cellular origin of GIST from the interstitial cell of Cajal (ICC) raises the question of whether these EGIST are truly an entity analogous to GISTs. It is not well known if extra-gastrointestinal stromal tumors (EGIST) originate from pacemaker cells outside of the GI tract or if mesenchymal cells have the ability to recapitulate the phenotype.

Sakurai *et al*. [12], published their results on the cytological, immunohistochemical, and genetic analysis of 5 omental mesenchymal tumors in 2001. They found all five tumors to be positive for CD117 and CD34 staining, while all were negative for smooth-muscle cell markers. More importantly, the authors reported finding KIT immunoreactive CD117 and CD34 cells within specimens of omentum [12]. These findings and those of Yamamoto *et al*., [13] underscore the fact that histologically, EGISTs have a similar appearance to GISTs, and that EGIST is a distinctive entity, different from leiomyosarcomas [4]. The most common mutation of the KIT gene occurred in exon 11 in Sakurai's and Yamamoto's experience [12,13]. According to Yamamoto [13], only 48% of his case tumors were positive for c-kit mutation (14 of 29 analyzed specimens). Of the EGISTs lacking detectable c-kit gene mutations, the author raised the possibility of an alternative oncogenic mechanism. Rubin *et al*., [14] demonstrated that, even in GISTs that lacked sequence mutations, KIT was highly phosphorylated.

Mitotic activity, cellularity and presence of necrosis have been found to be associated with worse outcomes. C-kit gene mutations were not found to correlate with prognosis in patients with EGISTs according to Yamamoto [13]. A high mitotic rate (>5/50 HPF) and a high Ki-67 labeling index (>10%) had a significantly poorer outcome. Reith *et al*. found a mitotic rate of >2/50 HPF, the presence of necrosis, and high cellularity to be useful in predicting biologic behavior in EGISTs, which tend to have an aggressive behavior similar to distal GI tract GISTs [15].

Of the omental EGISTs reported by Yamamoto, Sakurai, and Miettinen [4,12,13] (total of 28 cases), the mean diameter of the tumor was 15.35 cm. Only two patients with omental EGIST were younger than 40 years of age [4,13]. The follow up of the three patients with omental EGIST reported by Yamamoto was 6, 62, and 48 months; all three patients had no evidence of disease at end of follow-up [13]. In Miettinen's series, nine patients were followed, of which two died during follow up (one of colonic adenocarcinoma and another of unknown causes); six were alive and without evidence of disease at 2.34 years; one patient was alive and well at 8.5 years of follow up [4].

Of the reported omental and mesenteric EGISTs in four published series a total of 99 tumors were studied [4,12,13,15]. Of the 99 patients in these series, only 8 were under 40 years of age, none were younger than 30 years old; and only 5 were younger than 35 years old. Our patient's age is at the lower end of the age spectrum for the reported EGISTs. Of the 8 under 40 years of age patients with EGISTs, 6 were females. The youngest patient with an EGIST that we were able to identify in the literature was a 17 year old female with an abdominal wall EGIST [1]. GISTs are very rare in the pediatric population, but EGIST are even rarer. Of the 32 patients with omental or mesenteric EGISTs with follow up reported by Reith, 14 were alive and without evidence of disease with at least 7.5 months of follow up. Nine patients died of their disease at four months [15].

We achieved an R0 resection in our patient, but given the size and presence of necrosis in the tumor adjuvant STI-571 was started. There is no prospective data supporting the use of STI-571 in an adjuvant or neoadjuvant therapy after curative resection of GIST or EGIST. Yamamoto *et al*. suggests that the application of STI-571 could be a therapeutic strategy for EGISTs since they have kit alterations [13]. Todoroki *et al*. used STI-571 (300 mg/day orally) as adjuvant postoperative treatment in a 65-year-old female with a primary omental stromal tumor after R0 resection with a disease free follow-up at six months [16]. The American College of Surgeons Committee on Cancer (ACOSOG) is currently leading a phase II trial to test the benefit of adjuvant STI-571 with 400 mg/day for one year in patients after complete resection of high-risk tumors primary GISTs. The risk of recurrence after resection of a primary GIST is high. Conventional chemotherapy has proven ineffective against GIST (less than 10% response). The use of adjuvant STI-571 is based on the assumption of highest impact on residual microscopic disease, despite a negative margin of resection of the primary tumor [3]. STI-571 has demonstrated favorable response in more than half of patients with advanced and unresectable or metastatic GIST [17]. There has been reported resistance to STI-571 in patients with metastatic or recurrent disease, to which there are no good therapeutics currently [3].

## Conclusion

The existing data on EGIST is not sufficient to make a significant conclusion on the prognosis and survival of these patients, but certainly cellularity, mitosis and necrosis of the tumor appeared to be a prognostic factor [15]. While answers to the use of STI-571 in an adjuvant or even neoadjuvant setting are found, the management of patients with GIST or EGIST tumors at high risk of recurrence, such as ours, will be based on the clinical judgment of the treating physician and the availability of clinical trials.

## List of abbreviations

EGIST: Extra-gastrointestinal stromal tumor; GIST: Gastrointestinal stromal tumor.

## Competing interests

The authors declare that they have no competing interests.

## Authors' contributions

MC–S, SM, and MYA participated in the care of the patient. MC–S performed the literature review and drafted the manuscript. SM, BA, BG, AWT assisted in the review of the literature and in revising the manuscript. All authors read and approved the final manuscript.
